# Properties of Strandboard Panels Manufactured from Eastern Redcedar

**DOI:** 10.3390/ma2030926

**Published:** 2009-08-14

**Authors:** Salim Hiziroglu

**Affiliations:** Department of Natural Resource Ecology & Management, Oklahoma State University, Stillwater, Oklahoma 74078-6013, USA; E-Mail: salim.hiziroglu@okstate.edu; Tel. +1 (405) 744-5445

**Keywords:** strandboard, eastern redcedar, mechanical properties

## Abstract

This study evaluated physical and mechanical properties of experimental strandboard panels with random flake alignment manufactured from eastern redcedar (*Juniperus virginiana* L.) logs. Panels were made at two density levels of 0.65 g/cm^3^ and 0.78 g/cm^3^ using phenol formaldehyde adhesive applied at a rate of 8%. Mechanical properties including modulus of elasticity and modulus of rupture, and internal bond strength of the panels in addition to their thickness swelling characteristics were evaluated. As expected, mechanical properties of the samples improved with increasing panel density. Thickness swelling of the samples for 2- and 24-h water soaking test ranged from 6.32% to 18.41%. Both physical and mechanical properties of the panels showed acceptable results, comparable to those found in past studies using other species to manufacture similar types of product. Based on initial findings of this study it appears that eastern redcedar, which is an under-utilized invasive resource, has potential as a raw material for structural panel manufacture.

## 1. Introduction

Strand type composite panels such as oriented strandboard (OSB) are one of the engineered wood-based products most commonly used for structural purposes in construction, especially in the North American residential sector. Oriented strandboard was first produced in Canada in 1964, but it did not become a popular panel product for construction purposes until the 1980s. The number of OSB mills increased by more than 50% between 1990 and 1997 [1]. Today total OSB production in North America is approximately 20 million m^3^ and there are around 20 companies that manufacture OSB in the United States, Canada, and Europe. Forest products companies are aware of the need for more efficient utilization of raw materials with better technologies and in environmentally friendly ways. With this approach on management of forests, engineered wood composite products such as strandboard or OSB have gained an important role in the world market. A decline in plywood manufacturing in many countries due to limited large log supply and environmental concerns should also promote increased production of strand type panels in the future. In general, a simple spinning disk design is used to form the flake-containing mats. Strands of face and core layers of a typical mat are oriented in 90 degrees to each other, similar to the grain orientation of veneer in plywood manufacture and sequentially dropped on the conveyor belt so that mechanical properties of the panels can be enhanced. Structural panel products with randomly distributed strands were the predecessors of OSB. Usually OSB is manufactured from fast growing small trees. Oriented strandboard panel producers which are located in Western part of the USA use lodgepole pine logs with an approximate average 170 mm diameter at breast height (DBH) [1]. Southern pine from plantation thinning and soft hardwood species such as aspen, with an average DBH of 200 mm, are most widely used raw materials in the Southern states in the USA [1].

Eastern redcedar (*Juniperus virginiana* L.) is a widely distributed invasive species in Oklahoma and various other states, including Arkansas, Missouri, and Texas. The current area covered by eastern redcedar in Oklahoma is estimated to exceed 4.5 million hectares. It is projected that such a resource will cover 6.3 million hectares by 2013 [2,3,4,5]. Eastern redcedar population is growing at the rate of 380 hectares per day, resulting in a significant adverse impact on the environment [6]. If action is not taken it is predicted that problem caused by eastern redcedar invasion will cost $447 million by 2013 [6]. Currently large eastern redcedar trees are used for lumber manufacturing, while most of the small trees are burned in the field. This study addresses directly the development of value-added structural panel products from under-utilized eastern redcedar. The importance of this work lies in its potential to expand the use eastern redcedar in exterior structural composite panel manufacture, which may result in the development of an environmentally sound way to utilize this resource in Oklahoma. The overall objective of the study is to develop initial data to understand the characteristics of exterior type structural panels made from eastern redcedar logs in Oklahoma, without having any strand orientation. Therefore, this work addresses a major need to use invasive species as a raw material for strand type of panel manufacture and to test the properties of such panels to determine if they are similar to other panel products made from different raw materials.

## 2. Materials and Methods

**Figure 1:**
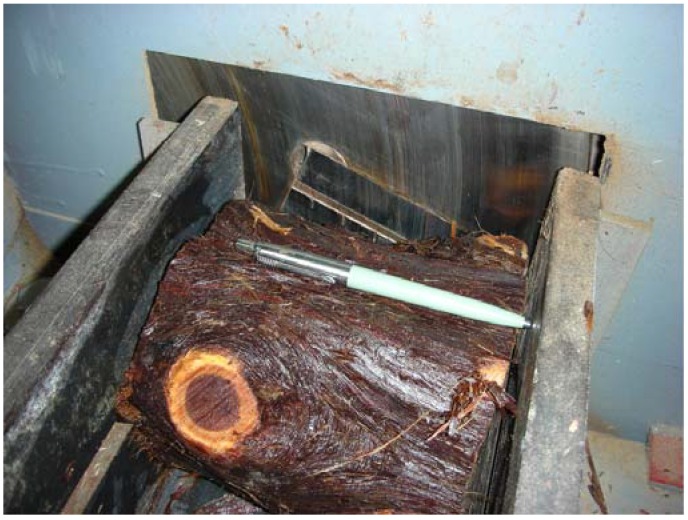
Strand generating process (courtesy of Forest Products Laboratory, Louisiana State University, Baton Rouge, LA, USA).

Five low quality eastern redcedar trees, with an average DBH of 150 mm were harvested in Southern Oklahoma. Logs were cut into 150 mm long sections and soaked into water prior to generating strands. A laboratory type disk flaker was employed to convert sections into strands, as illustrated in Figure 1. Strands were screened on a 10-mesh rotating sieve to eliminate oversize and undersize particles. Average strand size was 85 mm by 30 mm by 0.7 mm. The furnish was dried to 3% moisture content in a laboratory convection type oven prior to the adhesive blending process. An average of 8% liquid phenol formaldehyde adhesive based on oven-dry weight of strands with a solid content of 51% was sprayed to the material in a rotating drum equipped with a pressurized atomized gun. Single-layer hand-formed mats with random distribution of strands were manufactured on a frame with dimensions of 500 mm by 500 mm.

**Figure 2:**
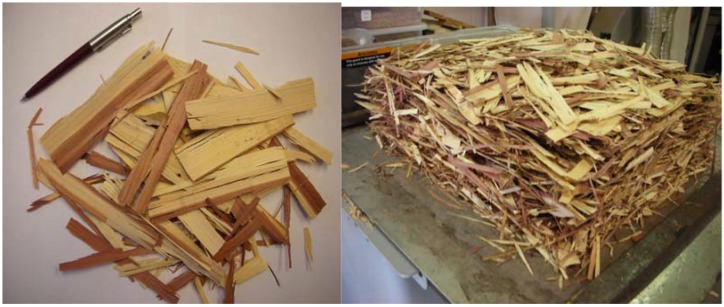

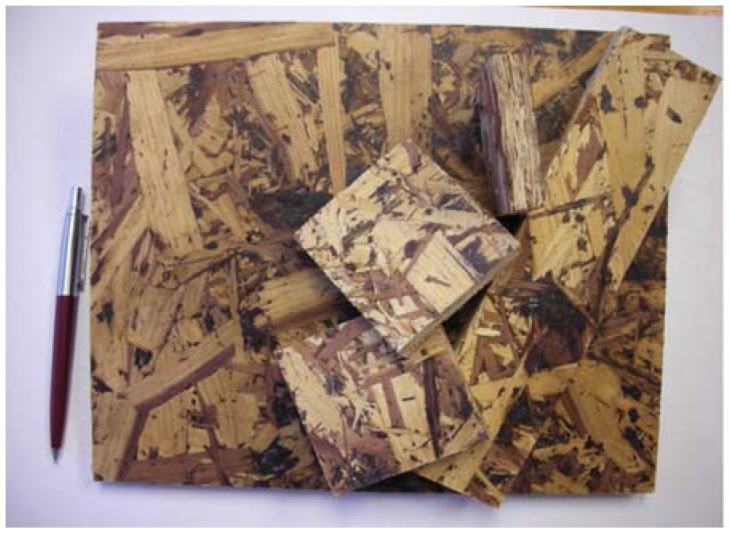
Eastern redcedar strands, unpressed mat, and finished samples.

The mats were compressed in a computer controlled press using 5.5 MPa pressure at a temperature of 160 °C for 8 minutes to a nominal thickness of 12 mm. Manufactured panels were then conditioned at a relative humidity of 60% and a temperature of 20 °C for two weeks before any tests were carried out. A total of 20 panels, 10 for each density level, namely 0.65 g/cm^3^ and 0.78 g/cm^3^. were produced for the experiments. Figure 2 shows strands, unpressed mat and finished samples. Specimens from conditioned panels were cut for various tests. Densities of whole panels and each individual test sample were also calculated. Modulus of elasticity (MOE), modulus of rupture (MOR), internal bond (IB) strength, and thickness swelling samples were prepared from each panel based on the ASTM D-1037 standard [7].

Three samples used for bending test were 290 mm by 76 mm by 12 mm. For internal bond strength test eight 50 mm by 50 mm specimens were cut from the panel. The dimensions of each sample were measured at an accuracy of 0.01 mm. Com-Ten Universal testing unit with 1,000 kg load cell capacity was employed for mechanical tests. Two 150 mm by 150 mm by 12 mm samples were cut from the center of each panels for thickness swelling. Average thickness of each samples was measured at five locations at an accuracy of 0.01 mm. Later the samples were submerged into distilled water for 2- and 24- h sequentially and their thickness was measured at each soaking level to determine their thickness swelling for two water exposures. Experimental sampling design is also given in Table 1.

The results of physical and mechanical properties were subjected to t-test analysis to determine any significant differences existed between mean values of properties of the samples as function of two density levels.

**Table 1:** Sampling design (values in parenthesis are standard deviation).

Panel type	Panel density (g/cm^3^)	Number of panels	Number of bending samples	Number of bending samples	Number of bending samples
A	0.65 ( 0.032)	10	12	48	12
B	0.78 (0.035)	10	12	48	12

## 3. Results and Discussion

Physical and mechanical test results of the samples are presented in Table 2. Internal bond (IB) strength is usually considered as an indicator of quality bond development within strand board. Average IB strength value was 0.81 MPa for the sample with 0.78 g/cm^3^ density. The other samples with lower density had 0.77 MPa as corresponding value, which was 5.2% lower than that of type-B panels.

The relationship between panel density and IB strength value was investigated in previous studies and it was concluded that there is well defined linear relationship between these two parameters with increasing panel density [8]. In another study the IB strength value of strand board made from larch was found as 0.72 MPa [9].

**Table 2:** Properties of the panels. (Values in parenthesis are coefficient of variation).

Panel type	Panel density (g/cm^3^)	Bending (MPa)		IB strength (MPa)	Thickness swelling (%)	
		MOE	MOR		2-hr	24-hr
A	0.65	2,845 (0.53)	17.5 (0.45)	0.77 (0.38)	6.32 (0.41)	15.24 (0.39)
B	0.78	3,331 (0.48)	21.3 (0.52)	0.81 (0.31)	8.32 (0.50)	18.42 (0.43)

**Figure 3:**
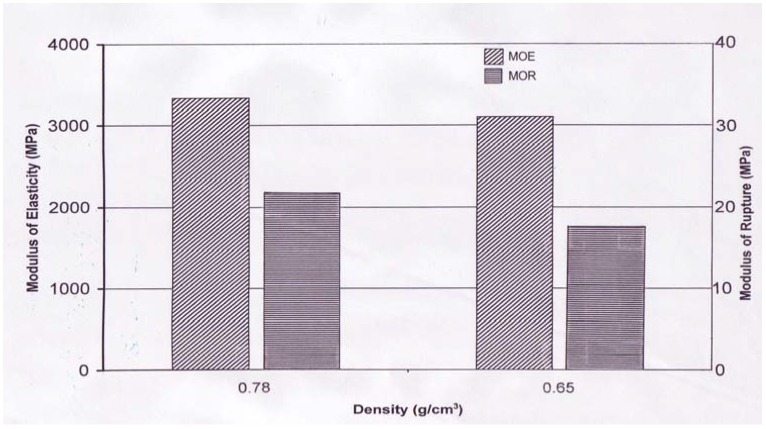
Bending properties of the panels.

**Figure 4:**
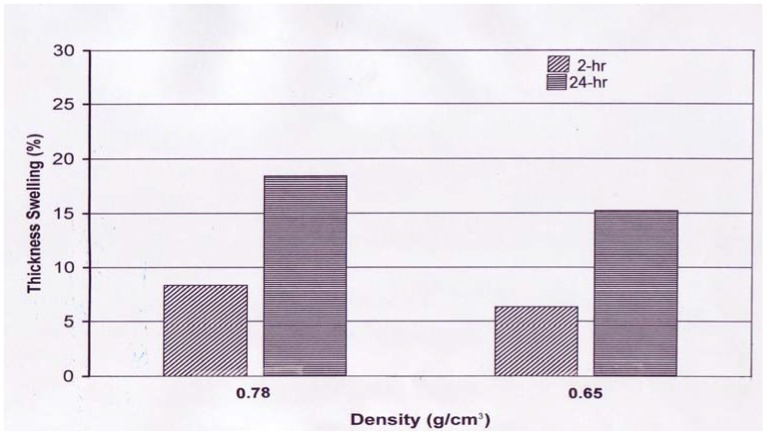
Internal bond (IB) strength values of the panels.

In addition to IB strength of the samples, results from a static bending test yielding MOE and MOR is a standard test to evaluate load resistance of structural type composites. Panel type-A (0.78 g/cm^3^) and -B (0.65 g/cm^3^) had 3,331 MPa, 2,845 MPa, 21.3 MPa, and 17.5 MPa for MOE and MOR values, respectively. Based on t-test, the bending properties of the samples with two different density levels showed significant difference from each other at 95% confidence. Panels with 0.78 g/cm^3^ density had 17.0% and 21.7% higher MOE and MOR values than those with lower density samples. Effect of panel density on internal bond strength was also observed [10,11,14]. Figure 3 and Figure 4 illustrate bending and IB properties of the samples.

Numerous studies have been carried out to evaluate relationship between bending characteristics and panels density of different types of wood composites including strand board. In one of these investigations it was found that strand board panels manufactured from Chinese tallow tree had 2,399 MPa MOE and 24.28 MPa MOR values [12].

Experimental bamboo strandboard panels in a previous work gave 4,000 MPa and 30.0 MPa for MOE and MOR values, respectively [13]. Mechanical properties of the samples determined in this work were comparable to those of past studies. As stated previously the main objective of this study was to obtain initial data on various basic properties of these experimental panels. If any orientation was given to the strand distribution in the mats during panel manufacture it would have been expected to have higher bending characteristics of such samples. Even with above mechanical properties it seems that eastern redcedar strand may have a potential to produce structural panels with acceptable mechanical properties.

Thickness swelling of the samples ranged from 6.32% to 18.42%. Panels with an average density of 0.78 g/cm^3^ showed 31.6% and 20.8% higher values for 2- and 24-hr water soaking than those of the panels with lower density. Higher thickness swelling values of composite panels with higher density related to their spring back characteristic have been discussed in various investigations [1,8]. Although no wax was used during panel manufacture overall dimensional stability of the panels are within acceptable range compared to the result of past studies [13,14]. Eastern redcedar had 3.8% oil in the heartwood with a dark red color, of course no classification was done to separate strands with heartwood and sapwood, and they were used in homogeneous mixture in panel manufacturing process [15]. However, still enhanced thickness swelling of the panels may possible related to oil content within the wood which may have acted a kind of wax resulting in better dimensional stability of the panels. Based on the statistical analysis all properties testes in the work showed significant difference as function of panel density.

## 4. Concluding Remarks

This study investigated some of the basic properties of strand board panels manufactured from eastern redcedar. Both mechanical and physical properties of experimental samples were determined as satisfactory and comparable to those found in previous works. These results suggest that such an underutilized species may have potential for the manufacture of structural composite panels. In further studies it would be desirable to make oriented strand board panels from 100% eastern redcedar strands and mixtures at different percentages with Southern pine strands. In addition to the properties tested in this study screw holding strength, linear expansion, and hardness of such panels would be tested to have a better understanding overall properties of the samples. Also, resistance of the panels against biological deterioration is planned to be investigated in the next phase of the study.
